# Chewing Difficulty Should be Included as a Geriatric Syndrome

**DOI:** 10.3390/nu10121997

**Published:** 2018-12-17

**Authors:** Jean Woo, Cecilia Tong, Ruby Yu

**Affiliations:** 1 Department of Medicine & Therapeutics, Faculty of Medicine, The Chinese University of Hong Kong, Hong Kong, China; rubyyu@cuhk.edu.hk; 2 CUHK Jockey Club Institute of Ageing, The Chinese University of Hong Kong, Hong Kong, China; ceciliatong@cuhk.edu.hk

**Keywords:** chewing difficulty, frailty, primary care, geriatric syndrome, oral health

## Abstract

Recent studies have noted an association between chewing difficulties and frailty. In a pilot survey of primary care needs of older people living in the community using automated methods, we examined the prevalence of chewing difficulties and the cross-sectional association with other geriatric syndromes, chronic diseases, and the use of hospital services. A brief multi-domain geriatric assessment was administered to 2259 men and women using a mobile device, the data uploaded to the cloud and analyzed. A total of 37.8% had chewing difficulties, which were associated with older age, poor vision, frailty, sarcopenia, memory complaints, low subjective well-being, incontinence, and stroke. The results suggest that chewing difficulties should be included as a geriatric syndrome and should be included in primary care screening of geriatric syndromes as well as chronic diseases.

## 1. Introduction

Chewing difficulties are more prevalent among older people, largely as a result of sub-optimal dental care or ill-fitting dentures, as well as a result of neurodegenerative conditions. It may be a reflection of lack of free or affordable dental services and have a significant impact on nutritional status [1,2,3], as well as increase long-term care needs and mortality [4]. Recent research from the field of dental and orofacial surgery has documented that edentulous older people were more frail than those with remaining teeth [5,6] and that older people with mild cognitive impairment or dementia have poorer oral health [7,8].

Difficulty in chewing food may have several underlying causes, which include the number of functioning teeth, periodontal health, masticatory force, sarcopenia, pain, xerostomia, as well as swallowing abnormalities (dysphagia). Together, these factors have been grouped under the umbrella of oral health, which impacts adequate food intake. Oropharyngeal dysphagia is now recognized to be a geriatric syndrome, particularly among frail older patients with physical as well as cognitive impairment, and is detected by a bedside swallowing test [9]. Dysphagia is also associated with frailty in the outpatient setting [10]. In the primary care setting, many health care systems deal with chronic diseases, while geriatric syndromes may be undetected if not actively elicited as patients may assume that these are natural accompaniments of old age. A quick first step screening to elicit these that does not require assessment by trained professionals may then be followed by comprehensive geriatric assessment and action by referral to appropriate health care professionals. Geriatric syndromes cover multiple domains, such as: Physical (sensory impairment and oral health, polypharmacy, incontinence, sarcopenia, frailty); functional (self-care ability and instrumental activities of daily living, IADL); and psychological (subjective well-being). This study examined chewing difficulties as part of a brief screen for geriatric syndromes as an overall indicator of possible problems with food intake.

Recent studies have documented an association between chewing difficulties and frailty [11,12,13]. While malnutrition is often associated with frailty in community studies [14,15], the association between chewing difficulties may not be entirely mediated by the nutrition pathway but may represent an independent risk factor for the onset of frailty [12]. Similarly, an association has been noted between oral health and cognitive impairment in a review of longitudinal studies examining oral health and cognitive decline [16]. A common inflammatory pathway has been postulated; however, studies showed no general agreement. It has been noted that poor oral health predisposes to a chronic low-grade inflammatory state through periodontal disease, which has been documented as a risk factor for cardiovascular diseases [17], as well as other chronic conditions with inflammation as the underlying etiology, such as the immune arthropathies, frailty, and sarcopenia [18].

Recently, the World Health Organization recommended an integrated primary care model for older people, consisting of multi-domain assessment through simple screening tools that does not require administration by health care professionals [19]. The aim of this study is to test the hypothesis that chewing difficulties form part of the geriatric syndrome.

## 2. Materials and Methods

### 2.1. Participants

This study uses data from a pilot primary care project for older Chinese people aged 60 years and over living in the community in Hong Kong, where basic information according to the World Health Organization Integrated Care for Older People, together with blood pressure, glucose, and body mass index (BMI) values were captured in community centers using an automated system and cloud technology, and then abnormalities dealt with by nurse-led teams by telephone as well as face-to-face meetings. The assessment was carried out from September 2016 to October 2017.

This was a community primary care project based in community centers run by non-government organizations in Hong Kong, to raise health literacy relating to common chronic diseases, such as hypertension and diabetes, as well as detection of unmet needs in relation to geriatric syndromes. Twenty-four centers participated on a voluntary basis, and each recruited approximately 100 members on a first-come-first-served basis. A tablet was used to record answers to questions, while blood pressure, blood glucose, body weight and height measurements were captured using equipment provided by two telecommunications companies and stored in their cloud. These data were then transmitted to a server in the Chinese University of Hong Kong and the data analyzed. The results of the questionnaire were sent to each participating center for feedback to the participants.

### 2.2. Methods

In a pilot study following this model using automated data captured through mobile devices among community living older people aged 60 years and over in Hong Kong, we examined the association between chewing difficulties and other commonly encountered geriatric syndromes, chronic diseases, and the use of hospital services.

The assessment questionnaire consisted of 5 categories, which included: (1) Socio-demographic characteristics (age, gender, education level, marital status, living arrangement, and disposable income); disposable income was based on the question ‘Do you have sufficient money to spend for daily living?’ rated on a 5-point Likert scale with 1 indicating ‘very insufficient’ and 5 as ‘very sufficient’; (2) geriatric syndromes (chewing difficulty, hearing impairment, vision impairment, frailty, sarcopenia, and subjective memorycomplaints, low subjective well-being, incontinence, problems with IADL) with answer ‘yes’ or ‘no’, or a scoring system for syndromes; any positive response of geriatric syndromes would trigger further questions or action; (3) presence of chronic diseases (hypertension, diabetes, high cholesterol, heart diseases, stroke, chronic obstructive pulmonary disease (COPD), and renal diseases); (4) number of medications; and (5) use of health services (hospitalization, specialist outpatient clinic (SOPC) visit, general outpatient clinic (GOPC) visit, and day care). The study was approved by the Survey and Behavioral Ethics Committee of the Chinese University of Hong Kong.

#### Details of Geriatric Syndromes

The assessment questionnaire for geriatric syndromes consisted of multiple domains, which use either single item questions or screening scales that are commonly used in the clinical care for the elderly.

Chewing difficulty. The presence or absence of chewing difficulty was based on answering ‘yes’ or ‘no’ to the question ‘Do you have any difficulty chewing?’, and if the participant had chewing difficulty, they were asked to indicate the reason/s for chewing difficulty based on the following: ‘Problems with dentures’, ‘no strength to chew’, ‘loose teeth or no teeth’, ‘painful gums or teeth’ or ‘dry mouth’.

Hearing impairment. The presence or absence of hearing difficulty was based on a single question: ‘Are you hearing clearly?’ Participants were asked to rate on a 5-point Likert scale, on which 1 indicated ‘very good’ and 6 indicated ‘very poor’.

Vision impairment. The presence or absence of vision difficulty was based on a single question: ‘Are you seeing things clearly?’ Participants were asked to rate on a 5-point Likert scale, on which 1 indicated ‘very good’ and 6 indicated ‘very poor’.

Frailty. Frailty is a state of decline in function reserves, which increases the risk of adverse health outcomes [20]. The 5-item FRAIL scale was used for screening frailty, which consists of five components: Fatigue: ‘Do you feel tired most or all of the time in the past 4 weeks?’; resistance: ‘Do you have any difficulty walking up 10 steps alone without resting and without aids?’; ambulation: ‘Do you have any difficulty walking 500–600 m alone and without aids?’; illness: ‘Do you have more than 5 illnesses?’; loss of weight: ‘Have you lost more than 5% of weight in the past few months?’ The presence of each characteristic was scored with 1 point (Yes = 1) and the absence of each characteristic was scored with 0 point (No = 0). The total FRAIL scale score ranges from 0 to 5, where a score of 0 represents robust, 1–2 represent pre-frail, and 3–5 represent frail health status [21,22].

Sarcopenia. Sarcopenia is characterized by the age-related loss of muscle mass and strength [23]. Sarcopenia was screened using the SARC-F questionnaire, which includes five components: Strength: ‘How much difficulty do you have in lifting and carrying 10 pounds?’ (none = 0; some = 1; a lot or unable = 2); assistance in walking: ‘How much difficulty do you have walking across a room?’ (none = 0; some = 1; a lot, use aids, or unable = 2); rising from a chair: ‘How much difficulty do you have transferring from a chair or bed?’ (none = 0; some = 1; a lot or unstable without help = 2); climbing stairs: ‘How much difficulty do you have climbing a flight of 10 stairs?’(none = 0; some = 1; a lot or unstable = 2); falls: ‘How many times have you fallen in the past year?’(none = 0; 1–3 falls = 1; 4 or more falls = 2). The total SARC-F score ranges from 0–10, where a score of ≥4 is predictive of sarcopenia [23,24].

Subjective memorycomplaints. Subjective memory complaints were assessed using the 5-item Abbreviated Memory Inventory for Chinese (AMIC). These questions focused on the subjective evaluation of memory problems [25,26]. The questions were: ‘Did you forget where you put your belongings?’, ‘Did you have problems recalling the names of your close friends?’, ‘Did you repeatedly forget what you intended to say during conversation?’, ‘Did you have difficulty in finding the right word to express yourself?’, and ‘Did you think that your memory was poorer when compared with others of similar age?’ Each ‘Yes’ response would receive a score of 1 (Yes = 1) and a ‘no’ response was each scored as 0 point (No = 0). The total AMIC score ranges from 0–5, with scores ≥3 as predictive of mild cognitive impairment.

Low subjective well-being. Subjective well-being refers to the subjective evaluation of a person’s overall quality of life [27]. Participants were asked 3 questions: ‘Are you satisfied with life?’, ‘Do you feel you have purpose and meaning in life?’, and ‘Indicate to what extent you consider yourself to be a happy person on a scale of 0 (unhappy) to 8 (happy)’. Low subjective well-being refers to the trigger of any of the following three items: Being dissatisfied with life, scoring ≤2 in the happiness scale, and having no sense of meaning and purpose in life.

Incontinence. Incontinence is a common problem in the elderly. In this current study, incontinence refers to either having urinary and fecal incontinence, which is the involuntary loss of urine or bowel contents. The presence or absence of this problem was determined with a single question: ‘Do you have problems with incontinence?’ with a ‘yes’ or ‘no’ response.

IADL. The IADL scale serves as a tool to assess independent daily living skills. IADL was assessed using 5 selected items extracted from the Chinese-version IADL adopted from the Lawton IADL scale [28,29]. Participants were asked if they were able to complete the following IADL tasks either independently or with occasional help or whether they were unable to do any of these in the past 3 months. The IADL tasks examined included the ability to use the telephone, shopping, food preparation, transportation, and the ability to handle finance. A score of 1 would be obtained if the participant is independent with each of the task, a score of 2 if help is needed to complete the task, and a score of 3 if the participant was unable to complete the task (Independent = 1; with help = 2; unable to do = 3). Participants scoring 2 or 3 in any of the items were classified as having an IADL problem, i.e., a total score > 5 indicates IADL problems.

### 2.3. Statistical Analysis

The baseline characteristics of participants with and without chewing difficulties were compared using the Chi-square test. Associations of socio-demographic factors (age, gender, education level, marital status, living arrangement, and disposable income), geriatric syndromes (hearing impairment, visual impairment, frailty, sarcopenia, subjective memorycomplaints, low subjective well-being, incontinence, IADL problems), chronic diseases (hypertension, diabetes, high cholesterol, heart diseases, stroke, COPD, renal diseases), the number of medications, and the use of health services in the past 12 months (hospitalization, SOPC visit, GOPC visit, day care) with chewing difficulties were analyzed individually using binary logistic regressions (Model 1). Odds ratios (ORs) with 95% confidence intervals (CIs) were reported. Subsequently, a binary multivariate logistic regression model (Model 2) containing all variables with a *p*-value < 0.1 in Model 1 was constructed. All analyses were carried out using the Windows-based SPSS Statistical Package (version 24.0; SPSS, Chicago, IL, USA), and *p*-values less than 0.05 were considered statistically significant.

## 3. Results

Among 2259 men and women, 38% had difficulty chewing. The commonest causes are listed in order of prevalence: Problems with dentures (17%), ‘no strength’ to chew (13%), loose teeth or no teeth (11%), painful gums or teeth (10%), and dry mouth (5%) (data not shown). Those with chewing difficulties were older, widowed, and had a lower education level and insufficient disposable income (Table 1). They also had a higher prevalence of heart disease and stroke and made greater use of hospital services.

Chewing difficulties were associated with all the domains of geriatric assessment. Logistic regression showed that older age (OR 1.67, 95% CI 1.28–2.19), having poor vision (OR 2.17, 95% CI 1.44–3.27), frailty (OR 2.21, 95% CI 1.61–3.04), sarcopenia (OR 1.52, 95% CI 1.13–2.06), memory complaints (OR 1.79, 95%CI 1.12–2.86), low subjective well-being (OR 1.38, 95% CI 1.09–1.75), incontinence (OR 2.06, 95% CI 1.12–3.80), and stroke (OR 1.76, 95% CI 1.14–2.72) are independent factors associated with chewing difficulties (Table 2). Participants with chewing difficulty had the highest odds ratio of being frail, independent of other co-variates.

## 4. Discussion

The results show that in the community setting, there is a high prevalence of chewing difficulties, which are independently associated with stroke, geriatric syndromes, and psychological well-being, highlighting the importance of detection of this condition in the primary care setting.

Poor oral health is not an inevitable part of aging, since good care throughout the life course could result in the maintenance of functional teeth to old age. Diseases that affect chewing, such as stroke, would unavoidably affect mastication. Difficulty with chewing may contribute to poor nutrition, which in turn predisposes to frailty and sarcopenia. However, in a subset of 1143 participants with available data on BMI, there was no significant difference in mean BMI between those with (24.93 ± 4.37 kg/m^2^), and without chewing difficulties (24.96 ± 3.90 kg/m^2^).

Poor oral health resulting in periodontal disease may predispose to frailty through a chronic inflammatory state, which has been postulated to account for it as a risk factor for cardiovascular diseases [17]. A chronic inflammatory state has also been postulated to be an underlying etiology of frailty and sarcopenia [30]. The finding of an association with cognitive impairment also supports the observation of an association between oral health and cognitive impairment, with a common inflammatory pathway postulated as the underlying etiology [16].

Therefore, among older people, chewing difficulties may not just increase the risk of poor nutrition, although this study did not collect extensive data on nutritional status to show this, but may indicate the presence of other geriatric syndromes as well and should be part of multi-domain assessment for older people in the community, as recommended by the World Health Organization [19]. Healthy aging emphasizes the preservation of function, and this study shows that chewing ability should be considered as an important component. A life course approach should also be adopted for oral health as for other health domains. As for chronic diseases and functional limitations, a social gradient is noted in chewing difficulties, illustrating the pervasive effect of social hierarchies on a wide range of health outcomes [31]. Social hierarchies and inequalities may impact oral health through choice of unhealthy lifestyles, in particular food choices and smoking habit. Unhealthy lifestyles may impact other aspects of aging in terms of multi-morbidity and frailty. Therefore, while those without chewing problems may be less at risk of reduced dietary intake, it does not necessarily follow that they will be healthier. Measures to promote oral health as a component of healthy aging would include provision of free or affordable dental care throughout the life course, as well as dietary adaptations to maintain optimal dietary intake to prevent frailty in the presence of chewing difficulties. The detection of chewing difficulties should be included as part of comprehensive geriatric assessment. Subsequent management would include dental assessment by an oral hygienist or dentist, the detection of xerostomia that may occur with some chronic diseases or medication, and a water swallowing test, which may include a video fluoroscopy swallowing study and flexible endoscopic evaluation of swallowing. Treatment would depend on the underlying cause, such as treatment for periodontitis or dealing with denture/loose teeth; advice regarding food preparations; and referral to speech therapists for swallowing exercises if indicated. Provision of free or low cost affordable dental care, accompanied by health promotion programs for oral health, would also be an important part of health policies for aging populations. Future cost-effectiveness modeling would be very useful in underscoring the need for such services, since it is likely to show economic benefits, a major driver to policy changes.

There are limitations in this study. The cross-sectional nature does not allow conclusions to be drawn regarding causation. We did not examine oral health in detail objectively but relied on self-reporting by participants. Although dysphagia may mediate the relationship between chewing and other geriatric syndromes, no swallowing assessment was carried out, since this was a pilot community screening initiative. There were no dietary intake data, anthropometric assessment other than BMI, and no objective measurements for the diagnosis of sarcopenia or cognitive impairment. Nevertheless, in screening for large populations in the primary care setting, the use of brief screening tools is a feasible option as the first stage in a step care approach. Those with abnormalities could then be assessed in greater detail by appropriate health care professionals.

## 5. Conclusions

The findings of this study support the proposal that chewing difficulties should be included as a geriatric syndrome. They also show the importance of detecting chewing difficulties as part of primary care for older people, as part of multi-domain health screening of geriatric syndromes that does not just focus on the detection of chronic diseases.

## Figures and Tables

**Table 1:** Socio-demographic characteristics, geriatric syndromes, chronic diseases, number of medications, and use of health services at baseline.

	Total Participants	With Chewing Difficulties	*P* ^1^
	*n* = 2259	*n* = 855	
	*n* (%)	*n* (%)	
**Socio-demographic characteristics**			
**Age**			
60–69 years	503 (22.3)	144 (16.8)	0.000
70–79 years	972 (43.0)	367 (42.9)	
80+ years	784 (34.7)	344 (40.2) ^3^	
			
**Gender**			
Women	1738 (76.9)	662 (77.4)	0.666
Men	521 (23.1)	193 (22.6)	
			
**Educational level**			
No education	633 (28.0)	289 (33.8)	0.000
Primary	1002 (44.4)	377 (44.1)	
Secondary	516 (22.9)	157 (18.4)	
Tertiary	107 (4.7) ^2^	31 (3.6) ^2,3^	
			
**Marital status**			
Married	1061 (47.0)	370 (43.3)	0.003
Single	103 (4.6)	31 (3.6)	
Widowed	958 (42.4)	403 (47.1)	
Divorced/Separated	137 (6.1) ^3^	51 (6.0)	
			
**Living arrangement**			
Not living alone	1470 (65.1)	539 (63.1)	0.122
Living alone	788 (34.9) ^2^	315 (36.9) ^2^	
			
**Disposable income**			
Far from sufficient/Insufficient	451 (20.0)	209 (24.4)	0.000
Just sufficient	1501 (66.4)	559 (65.4)	
Sufficient/More than sufficient	307 (13.6)	87 (10.2)	
			
**Geriatric syndromes**			
**Hearing**			
Very good/Good	1259 (55.7)	402 (47.0)	0.000
Fair/Not too well	909 (40.2)	398 (46.5)	
Poor /Very poor	91 (4.0) ^3^	55 (6.4) ^3^	
			
**Vision**			
Very good/Good	953 (42.2)	269 (31.5)	0.000
Fair/Not too well	1168 (51.7)	510 (59.6)	
Poor /Very poor	138 (6.1)	76 (8.9)	
			
**Frailty**			
Robust	809 (35.8)	206 (24.1)	0.000
Pre-frail (FRAIL = 1–2)	1040 (46.1)	412 (48.2)	
Frail (FRAIL ≥ 3)	409 (18.1) ^2^	237 (27.7)	
			
			
**Sarcopenia**			
Non-sarcopenic (SARC-F < 4)	1937 (85.7)	657 (76.8)	0.000
Sarcopenic (SARC-F ≥ 4)	322 (14.3)	198 (23.2)	
			
**Subjective memory complaints**			
AMIC = 0	137 (6.1)	27 (3.2)	0.000
AMIC = 1–2	436 (19.3)	112 (13.1)	
AMIC ≥ 3	1685 (74.6) ^2^	715 (83.7) ^2^	
			
			
**Low subjective well-being**			
No	1778 (78.7)	613 (71.7)	0.000
Yes	481 (21.3)	242 (28.3)	
			
**Incontinence**			
Self-controlled	1545 (68.4)	527 (61.7)	0.000
Occasional incontinence	651 (28.8)	289 (33.8)	
Incontinence	62 (2.7) ^2,3^	38 (4.4) ^2,3^	
			
**IADL problem**			
No problem, score = 5	1568 (75.5)	530 (67.6)	0.000
IADL problems, score > 5	508 (24.5) ^2^	254 (32.4)^2^	
			
**Chronic diseases**			
**Hypertension**			
No	782 (34.6)	289 (33.8)	0.525
Yes	1477 (65.4)	566 (66.2)	
			
**Diabetes**			
No	1628 (72.1)	605 (70.8)	0.280
Yes	631 (27.9)	250 (29.2)	
			
**High cholesterol**			
No	1631 (72.2)	599 (70.1)	0.076
Yes	628 (27.8)	256 (29.9)	
			
**Heart diseases**			
No	1911 (84.6)	694 (81.2)	0.000
Yes	348 (15.4)	161 (18.8)	
			
**Stroke**			
No	2157 (95.5)	798 (93.3)	0.000
Yes	102 (4.5)	57 (6.7)	
			
**COPD**			
No	2192 (97.0)	822 (96.1)	0.051
Yes	67 (3.0)	33 (3.9)	
			
**Renal diseases**			
No	2226 (98.5)	838 (98.0)	0.103
Yes	33 (1.5)	17 (2.0)	
			
**Number of medications**			
0	319 (14.2)	97 (11.4)	0.000
1–4	1407 (62.6)	511 (60.0)	
≥5	523 (23.3) ^2,3^	243 (28.6) ^2^	
			
**Use of health services**			
**Hospitalization**			
No	1735 (76.8)	625 (73.2)	0.001
Yes	523 (23.2) ^2^	229 (26.8) ^2^	
			
**SOPC visit**			
No	365 (16.2)	108 (12.6)	0.000
Yes	1892 (83.8) ^2^	746 (87.4) ^2^	
			
**GOPC visit**			
No	496 (22.0)	160 (18.7)	0.004
Yes	1763 (78.0)	695 (81.3)	
			
**Day care**			
No	2167 (96.0)	816 (95.4)	0.277
Yes	90 (4.0) ^2^	39 (4.6)	

Abbreviations: FRAIL, the 5-item FRAIL scale; SARC-F, the 5-item SARC-F questionnaire; AMIC, Abbreviated memory inventory for Chinese; IADL, Instrumental activities of daily living; COPD, Chronic obstructive pulmonary disease; SOPC, Specialist outpatient clinic; GOPC, General outpatient clinic. ^1^ *p*-values are based on chi-squared tests between elderly with chewing and without chewing difficulties. ^2^ Individual cells may not sum to total due to missing data. ^3^ Percentages may not add up to 100% due to rounding.

**Table 2:** Odds ratio (OR) of socio-demographic characteristics, geriatric syndromes, chronic diseases, number of medications, and use of health services at baseline.

	Model 1		Model 2	
	with Chewing Difficulties		with Chewing Difficulties	
	OR	95% CI	OR	95% CI
**Socio-demographic characteristics**				
**Age**				
60–69 years	Reference		Reference	
70–79 years	1.51	1.20–1.91	1.54	1.19–1.99
80+ years	1.95	1.53–2.48	1.67	1.28–2.19
				
**Gender**				
Women	Reference		-	-
Men	0.96	0.78–1.17	-	-
				
**Educational level**				
No education	Reference			
Primary	0.72	0.59–0.88	n.s	n.s
Secondary	0.52	0.41–0.67	n.s	n.s
Tertiary	0.49	0.31–0.76	n.s	n.s
				
**Marital status**				
Married	Reference			
Single	0.80	0.52–1.25	n.s	n.s
Widowed	1.36	1.13–1.62	n.s	n.s
Divorced/Separated	1.11	0.77–1.60	n.s	n.s
				
**Living arrangement**				
Not living alone	Reference		-	-
Living alone	1.15	0.96–1.37	-	-
				
**Disposable income**				
Far from sufficient/Insufficient	Reference			
Just sufficient	0.69	0.56–0.85	n.s	n.s
Sufficient/More than sufficient	0.46	0.34–0.62	n.s	n.s
				
**Geriatric syndromes**				
**Hearing**				
Very good/Good	Reference			
Fair/Not too well	1.66	1.39–1.98	n.s	n.s
Poor/Very poor	3.26	2.11–5.04	n.s	n.s
				
**Vision**				
Very good/Good	Reference		Reference	
Fair/Not too well	1.97	1.64–2.37	1.54	1.26–1.89
Poor/Very poor	3.12	2.17–4.49	2.17	1.44–3.27
				
**Frailty**				
Robust	Reference		Reference	
Pre-frail (FRAIL = 1–2)	1.92	1.57–2.35	1.53	1.22–1.91
Frail (FRAIL ≥ 3)	4.03	3.14–5.19	2.21	1.61–3.04
				
**Sarcopenia**				
Non-sarcopenic (SARC-F < 4)	Reference		Reference	
Sarcopenic (SARC-F ≥ 4)	3.11	2.44–3.97	1.52	1.13–2.06
				
**Subjective memory complaints**				
AMIC = 0	Reference		Reference	
AMIC = 1–2	1.41	0.88–2.26	n.s	n.s
AMIC ≥ 3	3.00	1.95–4.63	1.79	1.12–2.86
				
**Low subjective well-being**				
No	Reference		Reference	
Yes	1.92	1.57–2.36	1.38	1.09–1.75
				
**Incontinence**				
Self-controlled	Reference		Reference	
Occasional incontinence	1.54	1.28–1.86	n.s	n.s
Incontinence	3.06	1.82–5.15	2.06	1.12–3.80
				
**IADL problem**				
No problem, score = 5	Reference			
IADL problems, score > 5	1.96	1.60–2.40	n.s	n.s
				
**Chronic diseases**				
**Hypertension**				
No	Reference		-	-
Yes	1.06	0.89–1.27	-	-
				
**Diabetes**				
No	Reference		-	-
Yes	1.11	0.92–1.34	-	-
				
**High cholesterol**				
No	Reference			
Yes	1.19	0.98–1.43	n.s	n.s
				
**Heart diseases**				
No	Reference			
Yes	1.51	1.20–1.90	n.s	n.s
				
**Stroke**				
No	Reference		Reference	
Yes	2.16	1.45–3.22	1.76	1.14–2.72
				
**COPD**				
No	Reference			
Yes	1.62	1.00–2.63	n.s	n.s
				
**Renal diseases**				
No	Reference		-	-
Yes	1.76	0.88–3.50	-	-
				
**Number of medications**				
0	Reference			
1–4	1.31	1.00–1.70	n.s	n.s
≥5	1.99	1.48–2.67	n.s	n.s
				
**Use of health services**				
**Hospitalization**				
No	Reference			
Yes	1.38	1.13–1.69	n.s	n.s
				
**SOPC visit**				
No	Reference			
Yes	1.55	1.22–2.00	n.s	n.s
				
**GOPC visit**				
No	Reference			
Yes	1.37	1.11–1.69	n.s	n.s
				
**Day care**				
No	Reference		-	-
Yes	1.27	0.83–1.94	-	-

Abbreviations: OR, Odds ratios; CI, Confidence interval; FRAIL, the 5-item FRAIL scale; SARC-F, the 5-item SARC-F questionnaire; AMIC, Abbreviated memory inventory for Chinese; IADL, Instrumental activities of daily living; COPD, Chronic obstructive pulmonary disease; SOPC, Specialist outpatient clinic; GOPC, General outpatient clinic. Model 1: Crude. Model 2: Age, education level, marital status, disposable income, hearing, vision, frailty, sarcopenia, subjective memorycomplaints, low subjective well-being, incontinence, IADL problem, high cholesterol, heart diseases, stroke, COPD, number of medications, hospitalization, SOPC visit, and GOPC visit (logistic regression-forward: Wald). - Variables with a *p*-value < 0.1 in Model 1 were included in Model 2. n.s = Not significant in Model 2.
